# Intraspecific variation in body size does not alter the effects of mesopredators on prey

**DOI:** 10.1098/rsos.160414

**Published:** 2016-12-07

**Authors:** Austin J. Gallagher, Simon J. Brandl, Adrian C. Stier

**Affiliations:** 1 Rosenstiel School of Marine and Atmospheric Science, University of Miami, Miami, FL,USA; 2 Beneath the Waves, Inc., Miami, FL 33133, USA; 3 Smithsonian Environmental Research Center, Edgewater, MD, USA; 4 Department of Ecology, Evolution, and Marine Biology, University of California, SantaBarbara, CA 93106,USA

**Keywords:** predator, risk, functional response, fish

## Abstract

As humans continue to alter the species composition and size structure of marine food webs, it is critical to understand size-dependent effects of predators on prey. Yet, how shifts in predator body size mediate the effect of predators is understudied in tropical marine ecosystems, where anthropogenic harvest has indirectly increased the density and size of small-bodied predators. Here, we combine field surveys and a laboratory feeding experiment in coral reef fish communities to show that small and large predators of the same species can have similar effects. Specifically, surveys show that the presence of a small predator (*Paracirrhites arcatus*) was correlated with lower chances of prey fish presence, but these correlations were independent of predator size. Experimental trials corroborated the size-independent effect of the predator; attack rates were indistinguishable between small and large predators, suggesting relatively even effects of hawkfish in various size classes on the same type of prey. Our results indicate that the effects of small predators on coral reefs can be size-independent, suggesting that variation in predator size-structure alone may not always affect the functional role of these predators.

## 1. Introduction

Body size is a fundamental factor governing the effects of predators on prey [1]. Larger predators tend to have higher consumption rates due to their larger mouths and experience [2], and can develop highly adapted hunting strategies relative to their smaller counterparts [2,3]. Predator body size can also mediate the indirect effects of predators on prey; for example, a prey's perception of risk can increase with predator body size [4]. As ecosystems continue to experience decreases in predator body size due to the aggregate effect of disproportionate harvesting of large-bodied organisms [5] and the potential trophic release of smaller bodied predators (i.e. mesopredator release) [6], we will require a more detailed, mechanistic understanding of how predator body size mediates the effects of predators on prey.

Coral reefs are home to a diverse community of predators that vary in body size within and among species [7]. Surveys across gradients in exploitation pressure have demonstrated clear shifts in species composition and size structure within and among predator species [8], which has key implications for the rate at which energy flows throughout these nutrient poor ecosystems [9]. Experiments have demonstrated that predation is a key ecological force driving the population dynamics, biodiversity and community assembly of coral reef fishes [10–12], highlighting how the role of predation can shift with predator identity [13] and density [14]. However, the role of intraspecific variation in predator body size in mediating the effects of predators on prey remains understudied. Disentangling such size-dependent effects of predators on prey in coral reef fishes is critical to improving our understanding of how natural and anthropogenically induced variation in predator size affects the dynamics, structure and stability of coral reef communities.

In this study, we combine field observations and a laboratory experiment to quantify how intraspecific variation in the body size of a coral reef mesopredator (*Parracirrhites arcatus*) mediates its effect on prey fish communities in Moorea, French Polynesia. Specifically, we hypothesized that, if body size affects the direct and indirect effects of *P. arcatus* on prey, prey fishes would be (i) less likely to co-inhabit coral heads occupied by large *P. arcatus* than by small *P. arcatus*, and (ii) that attack rates of large *P. arcatus* under laboratory settings would be higher compared with small *P. arcatus*. If, however, body size has limited influence on the effects of *P. arcatus* on prey, we predicted no difference between small and large *P. arcatus* in both the field study (occupation of coral heads by prey fishes) and the laboratory study (survival of prey in tanks).

## 2. Material and methods

This study was conducted on a shallow back-reef on the north side of Moorea, French Polynesia (17°30′S, 149°50′W). The site is situated on the lagoonal edge of a barrier reef, which encircles the entire island and is defined by dense patches of reef and sand, with a depth range of 2–4 m. The arc-eye hawkfish (*P. arcatus*) is a voracious, diurnal and territorial ambush predator in this system, and is commonly observed perched upon the coral branches of *Pocillopora* spp. and *Acropora* spp. [15]. *Parracirrhites arcatus* are usually solitary with home ranges limited to approximately 5 m, resulting in the often sustained residence of singular individuals on the same coral head [16]. Their prey consists of a range of small fishes and invertebrates, including the two damselfish species *Chromis viridis* and *Dascyllus flavicaudus*. Both prey fish species are known to be extremely limited in their movements, commonly residing within 1 m of the coral head to which they recruited [17].

To assess the indirect effects of hawkfish of different sizes on their prey, we quantified patterns of hawkfish and their co-occurrence with other reef fishes by noting the presence of hawkfish and sympatric fish species, blue-green chromis (*C. viridis*) and the yellowtail damselfish (*D. flavicaudus*), on *Pocillopora eydouxi* coral heads along randomized 50 m belt-transect surveys parallel to the coastline. Surveys (*N* = 27) were conducted in the mornings (06.30–08.30) three times per week from 23 January to 13 February 2009. Previous work removing hawkfish from reefs in the same region showed that hawkfish presence decreased the density of small, coral-associated fishes [18]; however, the influence of size in these relationships remains unclear. Thus, if hawkfish were present, we visually estimated their standard length. The distribution of estimated fish sizes was bimodal; therefore, we classified hawkfish size categorically into two size classes: large (more than 4 cm TL) and small (less than or equal to 4 cm TL). While *P. arcatus* can grow to a maximum size of 20 cm, lagoonal systems are often characterized by the disproportional presence of small life-stages of reef fishes. Accordingly, during our transects no individuals approaching maximum size were observed. While both size classes are well beyond juvenile stages, individuals less than 4 cm TL may be characterized as sub-adults, while larger individuals are considered adults. We also estimated the abundance of other fishes within a 0.5 m lateral or vertical radius of the coral head. Each diver measured the dimensions of each coral head (length, width and height, in centimetres); no coral heads were surveyed more than once.

To estimate how variation in hawkfish size impacted hawkfish predation rates, we also conducted a laboratory feeding experiment from 14 February–10 March 2009, using live hawkfish and blue-green chromis (a potential prey species) from coral heads at the same study site where we performed the field surveys. Only co-occurring individuals from both species were selected for this study, and we never collected fish from coral heads that had previously been surveyed (they were flagged). Hawkfish (*N* = 22) and newly settled chromis individuals (*N* = 80, all fish sized approx. 0.5–2.0 cm) were collected from the field from 14–28 February 2009, using clove oil and hand-nets, and transported back to wetlab at the UC Berkeley Gump Marine Station (less than 2 km distance from collection site).

Hawkfish were initially separated into the same two size classes as described above; however, to better understand the effect of increasing body size, we restricted the size for the ‘large’ hawkfish to those larger than 9 cm TL (i.e. no individuals between 4 and 9 cm were used) (table 1). All fish were then placed in 25 gallon aquaria supplied with circulating seawater, and were separated by species, and starved and acclimatized for 24 h in the experimental arena. After 48 more hours (a total of 72 h post-collection), hawkfish were individually removed from their holding tanks, measured and categorized in terms of size, and placed into separate 1.5 × 1.0 m circular polypropylene tubs. Each tub contained circulating water and a sole piece of coral rubble (avg. size = 392 cm^3^) placed over a thin sandy substrate to serve as a temporary substrate for the predators and prey to use. All hawkfish immediately settled on the piece of rubble, and were allowed to acclimatize for 2 h. Chromis were then added to each tub in three density treatments for both size classes of hawkfish tubs: large hawkfish; low prey density (two individuals, *N* = 4), moderate prey density (four individuals, *N* = 3), and high prey density (six individuals, *N* = 5), and small hawkfish; low prey density (*N* = 4), moderate prey density (*N* = 2), and high prey density (*N* = 2), for a total of 20 trials (table 1). After each prey treatment was introduced, we monitored the abundance of chromis in each tub every morning at 08.00 over the next three days. 72 h after prey were introduced, we calculated *per capita* consumption rates for each hawkfish and treatment. Control tubs containing hawkfish and no prey (*N* = 4) and chromis without hawkfish (*N* = 2 for each treatment) were also created to examine natural survivorship (i.e. those still living after trials) under experimental conditions and we observed 100% survivorship in both cases.

**Table 1.** Experimental set-up for laboratory feeding assay using hawkfish (predator) and chromis (prey). The number of prey fish used is indicated in the parentheses for each of the three prey treatments. Numbers in the table represent the number of replicates for each prey treatment as it corresponds to the two predator identity treatments.

predator identity	high prey density (six fish)	medium prey density (four fish)	low prey density (two fish)
small hawkfish (less than 4 cm)	*N* = 5	*N* = 4	*N* = 3
large hawkfish (more than 9 cm)	*N* = 2	*N* = 2	*N* = 4

### 2.1. Statistical analyses

To test whether the presence of small and large hawkfish was correlated to the presence of prey fishes in coral heads, we performed a Bayesian Markov chain Monte Carlo (MCMC) generalized multi-response model with a binomial error structure [19]. We defined the presence or absence of prey fishes chromis and damselfish as a binary response variable, and defined the presence of either no hawkfish, small hawkfish (less than 4 cm TL), or large hawkfish (more than 4 cm TL) as the predictor variable. Owing to complete separation in the data, which can lead to unreliable estimates in regression models, we specified weakly informative Cauchy distributed priors on the fixed effects [20,21]. As residual variance is unidentifiable for binary responses, we fixed the residual variance to 1. We specified the chains to run for a total of 3 000 000 iterations, with a burn-in of 100 000 iterations and a thinning rate of 1000. Chain convergence was assessed using a visual assessment of chain trace plots (electronic supplementary material, 1). We used the posteriors to predict the probability (± 95% Bayesian credible intervals, CIs) of the two prey species being present on coral heads inhabited by small, large or no hawkfish. We used a binary response (presence versus absence of prey fishes) rather than abundance data (i.e. the number of prey fishes present), as we were primarily interested in indirect effects of hawkfishes on prey for the *in situ* part of the study. In other words, as it is difficult to disentangle direct consumptive effects in the field, we reasoned that coral heads inhabited by hawkfishes represent a ‘risky’ environment, avoided by prey species if suitable alternatives are present [4], and we sought to examine the effect of hawkfish size on this behaviour. The abundance data broadly corroborated the results based on the binary response and are provided in the electronic supplementary material, 2.

To assess the functional response of small and large hawkfish predators, we used Rogers random predator equation (RRP) for a Holling Type II response [20], which appropriately accounts for the effects of prey depletion throughout the experiment [21]. The response was modelled using maximum-likelihood estimation [22], with starting values extracted from previously published estimates on hawkfish functional response [15]. We extracted parameter estimates (attack rate *a* and handling time *h*) for both sizes of predators and calculated 95% CIs to permit comparison between small and large hawkfish. Owing to the short-term nature of our experiment, and the comparably low prey densities in the experiment, we focus largely on attack rate parameter estimates. All analyses were performed using the software R and the packages *MCMCglmm* [19] and *bbmle* [23].

## 3. Results

### 3.1. Field surveys

In total, we surveyed 67 coral heads, of which 47.3% were not occupied by hawkfish, 33.3% were occupied by small hawkfish, and 19.4% were occupied by large hawkfish. *Dascyllus aruanus* were present on 23 coral heads, while *C. viridis* were present on 16 coral heads. The two prey species co-occurred on 11 coral heads. The average size of the coral heads was 191.24 cm^3^ (± 45.6 s.e.). No coral head was inhabited by two *P. arcatus*.

**Figure 1.**
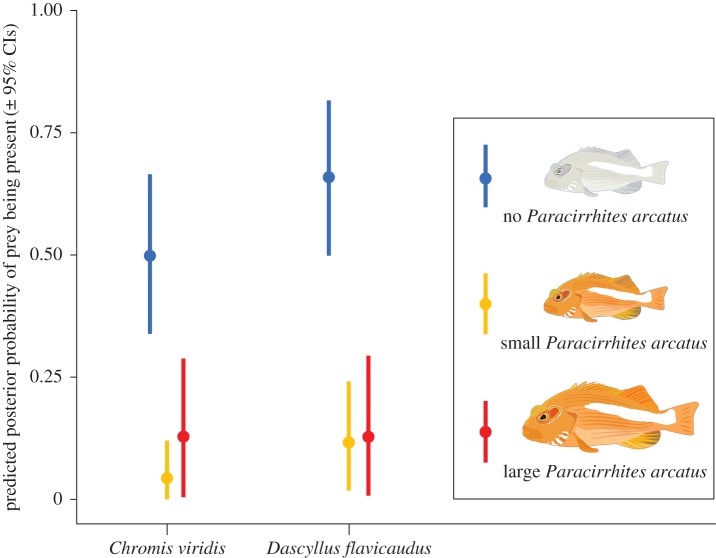
The effect of predator presence on prey fish presence. Caterpillar plots reflect the predicted posterior probability (± 95% credible intervals) of prey fishes in two species (chromis, damselfish) to be present on coral heads with either no hawkfish (blue), small hawkfish (orange) or large hawkfish (red). The plot shows that the likelihood of prey fish being present is smaller when hawkfish is present, regardless of the predators' size.

We found that the presence of hawkfish on coral heads correlated with a lower probability of chromis and damselfish being present on coral heads; however, there was no difference in the probability of prey fish presence between corals with small and large hawkfish, as there was extensive overlap in the estimated mean predicted posterior probability of either prey fish species being present (figure 1).

**Figure 2.**
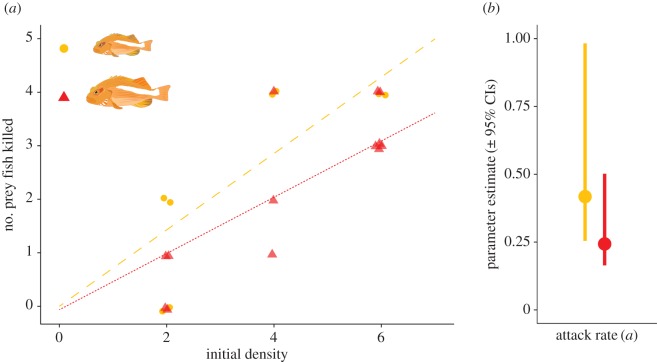
The functional response of small and large hawkfish under experimental settings. (*a*) Initial densities of prey fishes (chromis) are plotted against the number of prey fish consumed after 72 h. Treatments where small hawkfish were used as predators are shown in orange, whereas large hawkfish treatments are shown in red. As handling time *h* approaches 0 in both treatments, response curves are linear. (*b*) Attack rate estimates of small and large hawkfish. Estimates are similar for both types of predator, suggesting that size is of limited importance for the effect of hawkfish on prey. Statistically significant differences can be inferred where 95% CIs do not overlap.

**Figure 3.**
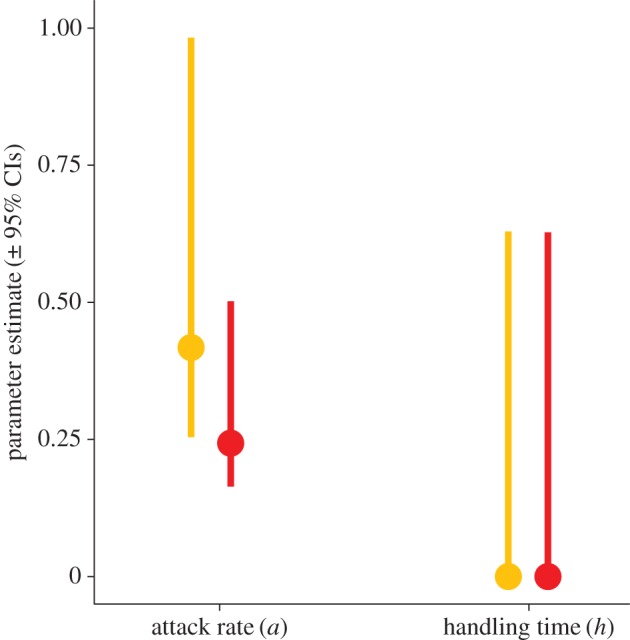
Parameter estimates for hawkfish functional response, including handling time parameter *h*. Treatments where small hawkfish were used as predators are shown in orange, whereas large hawkfish treatments are shown in red.

Likewise, our results suggest that the functional responses of small and large hawkfish do not differ under experimental settings (figure 2*a*,*b*). The parameter estimate of the attack rate *a* for small hawkfish was 0.418, with the 95% CI ranging from 0.254 to 0.983. This overlaps with the attack rate parameter estimate for large hawkfish, which was estimated as 0.243, with the 95% CI ranging from 0.164 to 0.502. Handling time *h* estimates were extremely low for both types of predators (less than 0.0001; figure 3), suggesting that experimental prey densities were too low to produce reliable estimates of handling time.

## 4. Discussion

Small predators may have disproportionally large roles in structuring food webs [24,25]. Yet, the role of intraspecific variation in body size has rarely been investigated in detail. We found that hawkfish occurrence was negatively correlated to the density of their prey species, but that these effects of small versus large hawkfish were statistically indistinguishable (figure 1). The indistinguishable estimates of small and large hawkfish attack rates corroborated our surveys, suggesting that size may not play a major role in determining the functional response of hawkfish. The size-independent nature of both direct and indirect effects of hawkfish on their prey suggests that natural variation in predator size (e.g. due to hawkfish condition and variable recruitment pulses) may not modify the effects of these small predators on their prey.

The results of our study require cautious interpretation due to several caveats. First, the laboratory results are obtained from a relatively small dataset, potentially influencing our ability to reveal differences in the attack rates (and handling times) of small and large hawkfishes. Capturing and transporting large hawkfish over 9 cm TL presented numerous logistical challenges and was deemed to impose significant stress to the animals, which affected our ability to bolster sample size in that treatment. Second, the size classes used for the experiment and encountered during the field surveys fall short of the maximum size of *P. arcatus*, which can grow up to 20 cm [26]. Therefore, it is possible that fishes approaching maximum size will differ from the smaller size classes investigated in the study. Third, only recently settled recruits of the prey species were used in this study. Given gape limitations of predatory fishes [27], it is reasonable to suggest that the impact of hawkfish on larger, adult prey will be determined by gape size. Nonetheless, our results convincingly show that in the investigated habitat (a shallow water lagoon that serves as a nursery for many species of coral reef fishes), the body size of *P. arcatus* appears to be of little consequence for the effect of this small predator on prey species during a critical life-stage.

The release of mesopredators from predation resulting from removals or losses of top predators has emerged as a key concept in conservation biology, highlighting the need to understand size-mediated effects of predators on prey [6]. On reefs where top predators are removed due to size selective harvesting [13], mesopredators often grow larger in size and live longer due to lower environmental threats [28]. Adult hawkfish are known to inhibit the recruitment success of their prey through both consumptive effects as well as indirectly by competing for predator-free space within their habitat [17]. Our results suggest that even small hawkfish can have a substantial effect on recently recruited prey species, both via direct and indirect effects. The notion that the attack rates of small mesopredators do not appear to scale allometrically is important for understanding how reefs may respond to changes in predator biomass and biological invasions. This emphasizes the need to test theoretical predictions linking body size to strength of predation. Recent work examining the effects of interspecific variation in predator body size demonstrated that large, mobile predators (such as reef sharks) occupy the same functional niche space as significantly smaller mesopredators on coral reefs [29]. Such deviations from expectations based on allometry suggest the link between body size and predation warrants further study on coral reefs. Changes in the size structure of marine food webs is a major consequence of extractive anthropogenic activities, which underscores the need to deepen our understanding of how shifts in body size are likely to affect ecosystem structure, function and resilience.

## Supplementary Material

ESM1: Trace chain plots for MCMC models, demonstrating chain convergence for both prey species, Chromis viridis and Dascyllus aruanus. (see attached figure)

## Supplementary Material

ESM2:Plots showing mean abundance of Dascyllus flavicadus and Chromis viridis as they relate to hawkfish presence (small hawkfish = < 4 cm; large hawkfish = > 4 cm) or absence (None) from the in-situ field surveys. Due to the zero-inflated nature of the data, we opted to treat all prey survey data as binary for modeling purposes in the manuscript. (see attached figure)

## Data accessibility

Our data are deposited at Dryad: http://dx.doi.org/10.5061/dryad.140p5 [30].
