# Supplementary figures and images for: Intraspecific variation in body size does not alter the effects of mesopredators on prey
Source: R Soc Open Sci. 2016 Dec 7;3(12):160414. doi: 10.1098/rsos.160414 (PMC5210675; doi:10.1098/rsos.160414)

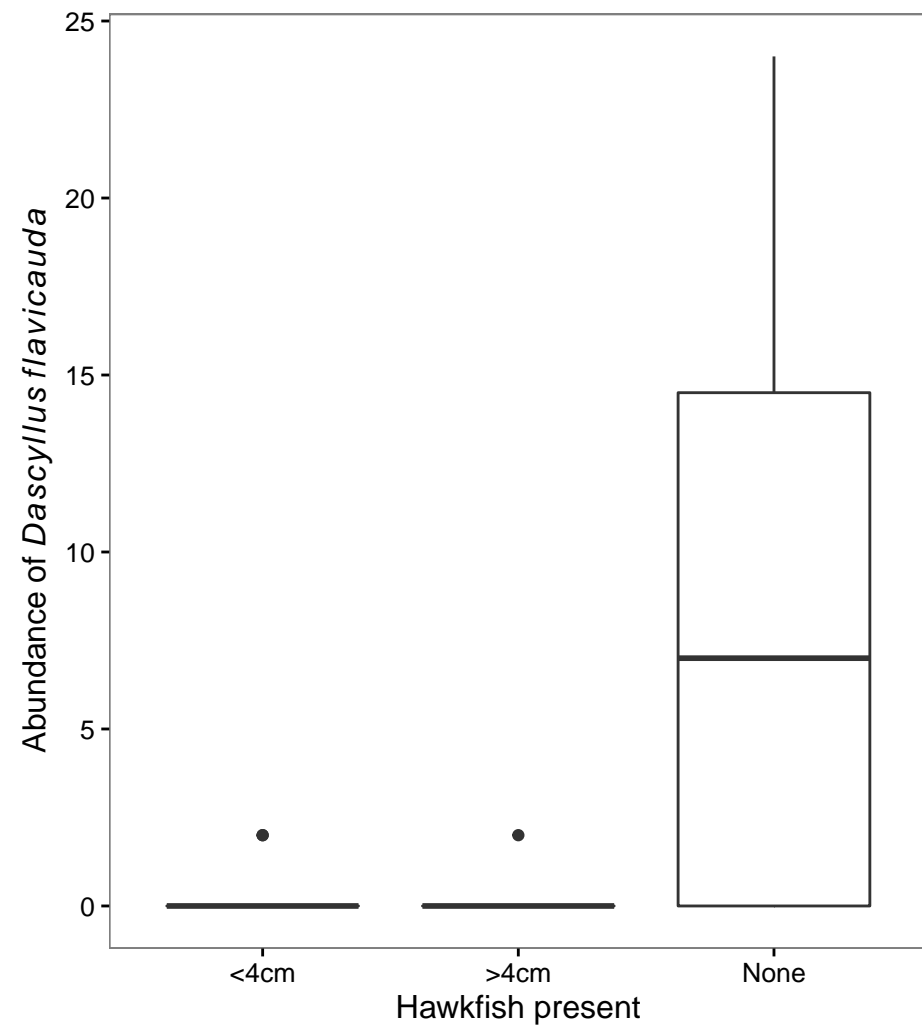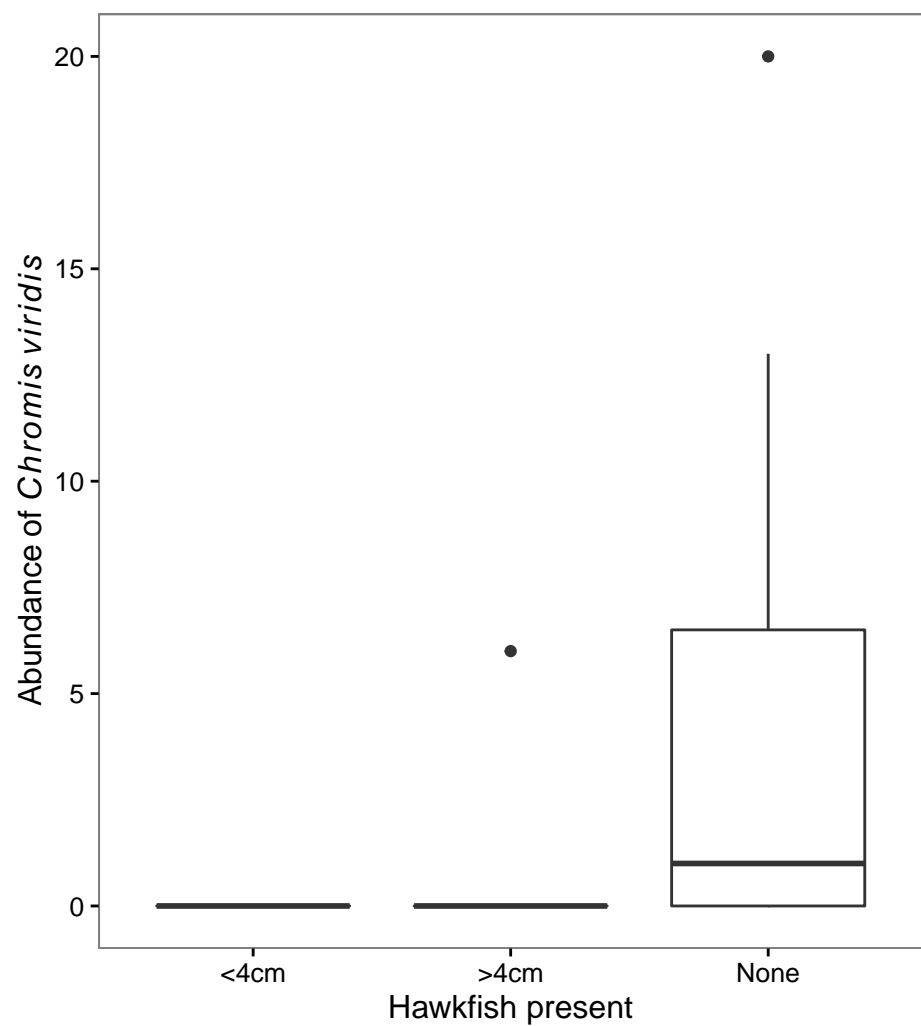

Supplement: ESM2:Plots showing mean abundance of Dascyllus flavicadus and Chromis viridis as they relate to hawkfish presence (small hawkfish = < 4 cm; large hawkfish = > 4 cm) or absence (None) from the in-situ field surveys. Due to the zero-inflated nature of the data, we opted to treat all prey survey data a [file rsos160414supp2.pdf]
